# Referred Trigeminal Facial Pain from Occipital Neuralgia Occurring Much Earlier than Occipital Neuralgia

**DOI:** 10.1155/2020/8834865

**Published:** 2020-08-24

**Authors:** Byung-chul Son

**Affiliations:** ^1^Department of Neurosurgery, Seoul St. Mary's Hospital, Seoul, The Catholic University of Korea, Republic of Korea; ^2^Catholic Neuroscience Institute, College of Medicine, The Catholic University of Korea, Seoul, Republic of Korea

## Abstract

We report a very rare case in which a patient believed to have auriculotemporal neuralgia due to the repeated recurrence of paroxysmal stabbing pain in the preauricular temporal region for four years developed occipital neuralgia, which finally improved with decompression of the greater occipital nerve (GON). The pain of occipital neuralgia has been suggested to be referred to the frontoorbital (V1) region through trigeminocervical interneuronal connections in the trigeminal spinal nucleus. However, the reports of such cases are very rare. In occipital neuralgia, the pain referred to the ipsilateral facial trigeminal region reportedly also occurs in the V2 and V3 distributions in addition to that in the V1 region. In the existing cases of referred trigeminal pain from occipital neuralgia, continuous aching pain is usually induced, but in the present case, typical neuralgic pain was induced and diagnosed as idiopathic auriculotemporal neuralgia. In addition, recurrent trigeminal pain occurred for four years before the onset of occipital neuralgia. If the typical occipital neuralgia did not develop in four years, it would be impossible to infer an association with the GON. This case shows that the clinical manifestations of referred trigeminal pain caused by the sensitization of the trigeminocervical complex by chronic entrapment of the GON can be very diverse.

## 1. Introduction

Occipital neuralgia is characterized by paroxysmal shooting or stabbing pain in the posterior part of the scalp with distributions of the greater and lesser occipital nerves (GON and LON) [1]. Although it has been suggested that the pain of occipital neuralgia can reach the frontoorbital (V1) area through trigeminocervical interneuronal connections in the trigeminal spinal nucleus [1], reports of referred trigeminal pain from occipital neuralgia are rare [2]. However, recently, pain referred to the facial trigeminal nerve distribution by occipital neuralgia due to GON entrapment has been reported [2–5]. Referral to the facial trigeminal distribution occurred not only in the V1 region but also in the V2 and V3 regions and even caused hemifacial sensory changes [2–5]. The referred trigeminal facial pain was immediately improved by decompression of the GON. In the previous reports, the nature of the referred facial trigeminal pain caused by GON entrapment showed continuous aching pain and occurred months after the symptoms of occipital neuralgia [2–5]. However, the authors experienced a very rare case where referred facial trigeminal pain appeared in the form of severe paroxysmal stabbing pain in the preauricular temporal area rather than aching pain. In addition, this facial pain occurred years before the symptoms of occipital neuralgia and continued to recur. These aspects made the diagnosis very difficult.

The natural course of untreated occipital neuralgia due to chronic entrapment of the GON remains unknown [5]. The GON originates from the medial branch of the dorsal rami of the second cervical nerve root [6–9]. Therefore, the GON constitutes the main sensory afferent nerve through the C2 root, and this afferent input is directly relayed to the C2 dorsal horn [10–12]. The etiology of primary occipital neuralgia is mostly unknown [2]. However, entrapment of the GON at its piercing point of the tendinous aponeurotic attachment of the trapezius at the superior nuchal line is known as the most common cause [2, 8, 13]. The chronic continuous and noxious afferent input of occipital neuralgia caused by GON entrapment seems to be associated with sensitization and hypersensitivity of the second-order neurons in the trigeminocervical complex, a population of neurons in the C2 dorsal horn characterized by receiving convergent input from dural and cervical structures [10–12].

## 2. Case Report

**Figure 1:**
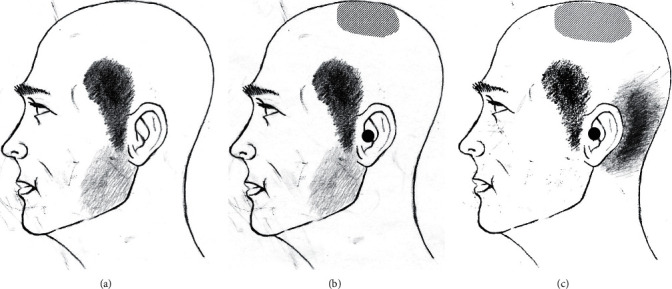
Schematic diagram demonstrating the distribution and characteristics of pain over the years. (a) The gray areas over the left preauricular temporal area indicate the distribution of paroxysmal lancinating pain that started in 2014. Pain initially began as a persistent aching pain in the left molars and gums (light gray area). (b) In 2016, paroxysmal pain occurred in the left preauricular temporal region, and at the same time in the left vertex (obliquely hatched area) and the left deep ear (black circle). (c) It was not until 2018, four years after the occurrence of paroxysmal pain, that the stabbing pain in the retroauricular occipital area (transversely hatched area) occurred as pain in the left temporal region (gray area), left vertex (obliquely hatched area), and left deep ear (black circle).

In December 2014, a 78-year-old female presented with severe paroxysmal stabbing pain in the front of the left ear and anterior temporal region. The pain initially began with persistent aching pain in the left molars and gums, and severe paroxysmal stabbing pain occurred at 5 to 10 second intervals over a day or two. Paroxysmal pain continued to occur even when she was resting and was more severe when she moved her jaw or spoke or lightly touched the front part of her left ear and temporal region (Figure 1(a)). The left facial pain was severe (7 to 8 out of 10 on the numerical rating scale (NRS-11)). Physical examination showed no changes in facial sensation and no abnormal findings in the cranial nerve, but pulsatile pain and tenderness of the superficial temporal artery in the anterior temporal region were observed.

Under the preliminary diagnosis of trigeminal neuralgia or temporal vasculitis, she was prescribed carbamazepine and gabapentin and consulted with the Rheumatology department. The laboratory examinations showed normal findings, including an elevated sedimentation rate (ESR) of 6 mm/hr. Magnetic resonance imaging (MRI) scans suspected for trigeminal neuralgia but did not show vascular compression or abnormal enhancement of the trigeminal nerve. After about 10 days, her severe pain gradually disappeared with medication and no special sequelae were observed.

Nine months later, in September 2015, her severe pain suddenly recurred without any special event. As in the past, the paroxysmal stabbing pains in the preauricular and temporal areas continued. Especially when she moved her chin, the pain in her lower gum was so severe that she could hardly eat. Nonsteroidal anti-inflammatory drugs (NSAIDs) were ineffective, and the pain did not decrease with gabapentin 1600 mg/day, pregabalin 450 mg/day, tramadol 450 mg/day, and amitriptyline 30 mg/day. Her pain was so severe (NRS-11, 7-8/10) that she had to be hospitalized for pain control three weeks after she became sick. The neurological examination showed no abnormalities, and severe tenderness of the temporal area was present, but no swelling was observed. IR-codon® (40 mg/day) added to the anticonvulsants had little effect on pain control, and only an intramuscular injection of diclofenac sodium could reduce the pain by half for three hours. There were no abnormal findings on laboratory tests including ESR and C-reactive protein (CRP). No abnormal findings of the temporal artery and trigeminal nerve were observed in color-duplex ultrasonography and contrast-enhanced MRI. No sinus, ear, tooth, and temporomandibular joint (TMJ) problems were found upon in otolaryngology and dental consultations.

Unusually, the patient reported the paroxysmal pain was as if the temporal artery's pulse was beating. The rheumatologist did not conduct a biopsy of the temporal artery because there were no signs of inflammation and no imaging problems. Excluding the possibility of temporal arteritis, we tentatively determined that it is rare but an auriculotemporal neuralgia for pain distribution. Blocks with 1% lidocaine (3 ml) were used near the temporal artery. Pain relief was observed for about one hour after the injection, but the pain in the temporal region and gums continued. Even when the patient was hospitalized for 10 days and visited the hospital two weeks after her discharge, she said she was sick enough to have trouble sleeping at night. Her severe pain gradually diminished after a month and a half. No more medication was needed.

In September 2016, the same pain suddenly recurred. This time, the patient had been tired for several days before the pain occurred. The pain also occurred spontaneously in the preauricular temporal area, and she said that the aching pain occurred surprisingly suddenly. This time, paroxysmal pain occurred in the left vertex and deep in the left ear at the same time as the left temporal region (Figure 1(b)). No ear abnormalities were identified in the otolaryngology consultation. A temporal local block and mandibular nerve block were ineffective. Again gabapentin, carbamazepine, tramadol, and amitriptyline were administered, and the pain improved after two weeks. A month later, it reappeared for about two weeks and improved.

In November 2017, she visited our outpatient clinic after receiving treatment at another hospital for the same pain in the preauricular temporal area and vertex. She received similar medications and nerve blocks in other clinic, and resection of the superficial temporal artery was recommanded but she did not have surgery. After about three weeks of medication, the pain disappeared spontaneously. Two months later, in January 2019, pain of the same nature recurred to the left temporal and gum region, inside the ear canal, and parieto-occipital vertex (Figure 1(b)). Her pain was so severe (NRS-11, 6-8/10) and unremitting that she was hospitalized for a week with regular diclofenac sodium injections. Neurological and laboratory testing and MRI were performed again, but no abnormal findings were found. The pain subsided spontaneously after three weeks after discharge.

The patient experienced no pain for the next seven months, but in August 2018, her severe paroxysmal pain recurred again in the left temporal, parieto-occipital vertex, and inside the left ear. The same stabbing pain occurred in the same manner as before, and pain occurred when she moved her head. Specifically, this time, the pain also occurred in the occipital area behind the ear, in the distribution area of the greater occipital nerve (Figure 1(c)). With the possibility of referred trigeminal facial pain from occipital neuralgia in mind, an occipital nerve block (ONB) was performed using 2 ml of 1% lidocaine for the left GON. The effect of the ONB was dramatic for two hours. In addition to the pain in the occipital and vertex area, the paroxysmal pain in the anterior temporal region and deep ear area was significantly improved. She estimated that the pain was reduced by 90% (9/10 for the NRS-11 scale improved to 2-3/10 after one hour). However, the effect of the ONB disappeared completely after two hours and she became ill again. Transient hypesthesia due to ONB was observed in the occipital and parietal vertex area and the GON distribution area but not in the temporal region.

No abnormalities were detected in the computed tomographic (CT) scan of the cervical spine performed to check for structural lesions in the path from the C2 nerve root to the GON. Two ONBs were performed after admission, and pain reduction in the same occipital and preauricular areas was repeatedly confirmed. Considering the intractability and the possibility of referred trigeminal pain of occipital neuralgia, GON decompression was explained to the patient and her caregivers, and informed consent was obtained.

**Figure 2:**
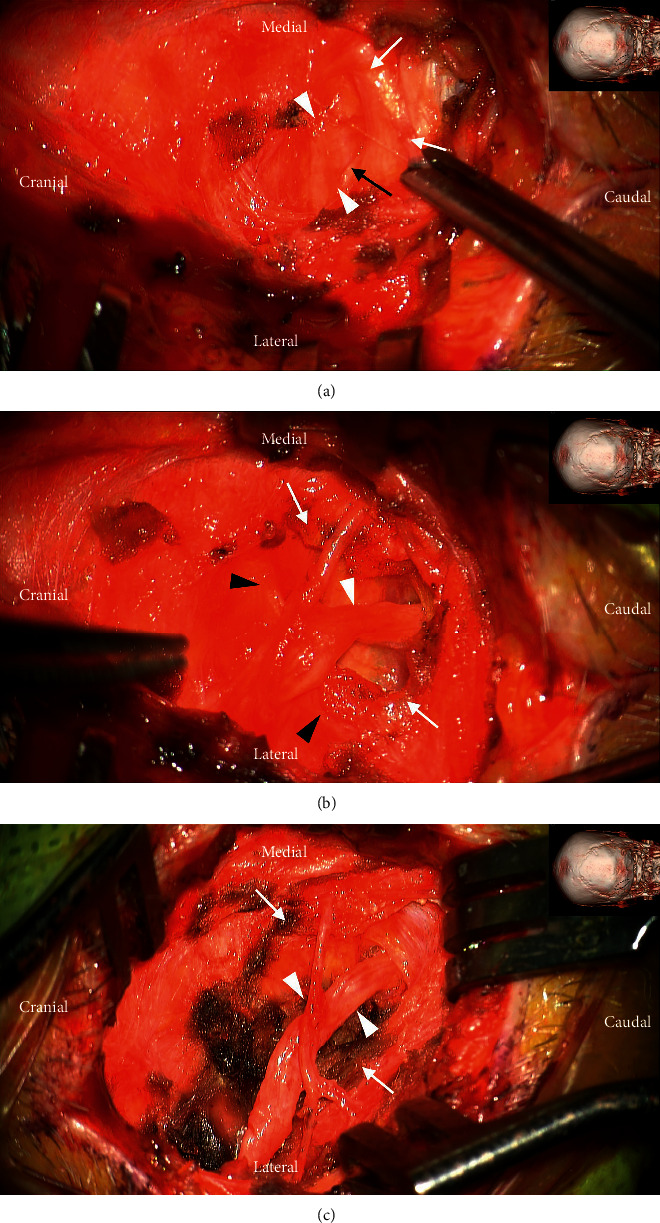
Intraoperative photographs during the decompression of the greater occipital nerve (GON). (a) Intraoperative photograph showing severe entrapment of the GON (white arrowheads) by the aponeurotic fibrous attachment of the trapezius (white arrows) at the superior nuchal line. The maximal compression point (black arrow) is seen by lifting the trapezial aponeurotic edge. The inset shows the direction of the photo and the location of the incision. (b) Intraoperative photograph after decompression of the left GON (white arrow heads) with the division of the trapezial aponeurosis (white arrows). Even after decompressing the upper side, the GON was still depressed by the lower fascial aponeurosis (black arrowheads). (c) Intraoperative photograph after complete decompression of the left GON (white arrowheads). Its proximal and distal courses are addressed.

A 4 cm-sized linear incision was made obliquely along the course of the left GON. The incision was then developed over the trapezius fascia. The GON was found to be severely entrapped by the aponeurotic arch of the trapezius, showing an indentation of chronic entrapment (Figures 2(a) and 2(b)). After the circumferential release of the GON from the tendinous aponeurotic arch, the proximal and distal courses of the GON were dissected. A small tributary of the occipital artery was found to constrict the distal course of the GON, which was cut off, resulting in complete decompression of the left GON (Figure 2(c)). The patient's pain attacks persisted for two days after surgery, and then the instances and intensity of pain decreased from day three. From seven days after surgery, the number of paroxysmal pain attacks decreased by more than 80%, and numbness was observed in the left occipital area. A month later, the paresthesia of the left occipital disappeared, and there was no more painful paroxysm of the left temporal lateral temporal region, inside the ear canal or the occipital area. The patient's pain attack no longer occurred until a year and a half after surgery.

## 3. Discussion

### 3.1. Atypical Manifestation of Occipital Neuralgia: Referred Ipsilateral Trigeminal Pain

Occipital neuralgia is defined as paroxysmal, shooting, or stabbing pain in the posterior part of the scalp, in the distribution(s) area of the greater, lesser, and/or third occipital nerves [1]. Occipital neuralgia pain has been suggested to be referred to the frontoorbital (V1) area through trigeminocervical interneuronal connections in the trigeminal spinal nucleus [1], but reports of such cases are very rare [2]. However, in occipital neuralgia, referred pain in the ipsilateral facial trigeminal region has been reported to occur in the V2 region as well as in the V2 and V3 regions and the frontal-orbital (V1) region [2–5]. Occipital neuralgia caused by chronic GON entrapment has been reported to cause not only facial pain but also hemisensory changes throughout the ipsilateral face [2, 3]. These findings provide clinical affirmation of the existence of trigeminal/cervical convergence and hypersensitivity [2, 10, 11].

The trigeminocervical complex (TCC) is a population of neurons in the C2 dorsal horn that receive convergent input from facial skin corresponding to the trigeminal nerve and cervical structures innervated by the high cervical C1-3 roots [10–12]. Convergence of nociceptive afferents and sensitization of TCC neurons have clinical correlates, including hypersensitivity and the spread and referral of pain frequently seen in patients with primary headaches such as migraines [10, 11]. Patients with primary headaches often report pain not only involving the frontal head innervated by the trigeminal nerve but also involving the occipital region innervated by the GON [10]. The sensitization of central nociceptive neurons in the TCC takes place in response to strong dural noxious input seen in secondary headache syndromes such as meningitis, subarachnoid hemorrhage, and experimental headaches [12]. Chronic compression and adhesion of the GON by the occipital artery and its constriction by the aponeurotic fibrous aperture of the trapezius muscle can cause chronic occipital neuralgia [2–5, 13]. The chronic noxious afferent input of occipital neuralgia may lead to central sensitization of the TCC, resulting in referred hemifacial trigeminal pain [2, 4, 10, 11].

### 3.2. Preauricular Temporal Pain and Deep Ear Canal Pain as Referred Pain

Although initially accompanied by pain in the lower and upper gums, specifically, the main pain in this patient occurred in the temporal region in front of the ear. Due to this unusual distribution of pain, it was considered rare but auriculotemporal neuralgia (ATN). The auriculotemporal nerve is a terminal branch of the mandibular nerve (third division of the trigeminal nerve). ATN neuralgia is characterized by paroxysmal attacks that are strictly unilateral and lancinating, moderate to severe pain on the preauricular area often spreading to the ipsilateral temple [14, 15]. The pain in this patient showed typical neuralgic pain, and the location of pain was the same as that of the ATN distribution. However, blocks around the ATN were not effective for pain in the preauricular temporal area, and ONB performed after the occurrence of occipital neuralgia was effective for pain in the preauricular temporal area. It is difficult for us to explain this phenomenon. We speculated that the referred pain in the trigeminal distribution could to be reduced by blocking the area that caused the referred pain.

Another peculiarity was the pain inside the ear canal that accompanied the occurrence of the typical occipital neuralgia in the temporooccipital area. The pain inside the ear canal was also typical neuralgic pain and was present in the auditory canal not the eardrum. It was not related to any activities, such as drinking, sneezing, or coughing. The anterior wall of the ear canal is innervated by the ATN, and the posterior wall is made by the auricular branch of the vagal nerve (ABVN) [16]. It is difficult to identify which of the two nerves was involved. However, it was reasonable to think of the pain in the deep ear as pain referred to the ATN, a branch of the trigeminal nerve, because typical pain occurred in the ATN of the preauricular temporal area. This was confirmed by temporary improvement of the deep ear pain with the ONB.

### 3.3. Entrapment Neuropathy of the Greater Occipital Nerve

The GON originates from the medial branch of the dorsal rami of the second cervical nerve root [7–9]. It also receives a branch from the dorsal rami of the C3 nerve. It ascends through the semispinalis muscle and then runs rostrolaterally before emerging into the scalp by piercing the aponeurotic fibrous sling between the trapezius and the sternocleidomastoid near their occipital attachment to the superior nuchal line [17, 18]. This aperture (trapezial tunnel) is a common site of entrapment of the GON [8, 17, 18]. At this point, the GON travels with the occipital artery, running form the anterolateral direction beneath the splenius capitis to supply the integument of the scalp as far anterior as the vertex of the skull [6–9]. After piercing the aponeurotic sling between the trapezius and the sternocleidomastoideus, in contrast to common findings in other peripheral nerves, the GON widens toward its course to the periphery [6]. This finding is regarded as relevant to the entrapment of the GON, as widening of the nerve makes it susceptible to entrapment especially in the firm trapezius muscle aponeurosis [6].

We think that the term “entrapment” is more appropriate than simple compression of the GON. The term “entrapment neuropathies” refers to isolated peripheral nerve injuries that occur at specific locations where a nerve is mechanically constricted in a fibrous or fibro-osseous tunnel or deformed by a fibrous band [19].

### 3.4. Difficulty in Diagnosing Referred Trigeminal Pain due to Chronic Entrapment of the GON

The natural course of occipital neuralgia due to chronic entrapment of the GON remains unknown. As shown in the present case, chronic entrapment of the GON can cause pain in the V2 and V3 dermatomes. In past cases [2–5, 13], continuous aching pain was induced, but in the present case, typical neuralgic pain was induced and diagnosed as idiopathic ATN neuralgia. Moreover, facial pain preceded occipital pain four years earlier. If the neuralgic pain, which recurred every year for several years, was not accompanied by occipital pain later, it would not be possible to infer GON involvement. Also, unlike trigeminal neuralgia, in which neurovascular contact can be confirmed by MRI, it is difficult to determine the degree of entrapment of the GON within the small trapezial canal. Thus, when unexplained facial pain is accompanied by typical paroxysmal stabbing pain in the occipital area, we recommended confirming that there is no structural lesion through a detailed MRI examination and checking whether facial pain decreases through repeated ONBs.

## 4. Conclusions

We report a case of occipital neuralgia due to chronic GON entrapment, which was preceded by neuralgic pain in the face. The pain in the distribution of the ATN, the third branch of the trigeminal nerve, was very severe and recurred, and occipital nerve involvement was not suspected until the occurrence of occipital neuralgia. This case shows that the clinical manifestations of referred trigeminal pain caused by the sensitization of the trigeminocervical complex by the chronic entrapment of the GON can be very diverse.
